# Supplementary material for: Universal screening for HCV infection in China: An effectiveness and cost-effectiveness analysis
Source: JHEP Rep. 2024 Jan 11;6(4):101000. doi: 10.1016/j.jhepr.2024.101000 (PMC10933547; doi:10.1016/j.jhepr.2024.101000)
Supplement: Multimedia component 1 [file mmc1.pdf]

Universal screening for HCV infection in China: An effectiveness and cost-effectiveness analysis

Hua Zhou, Mengxia Yan, Datian Che, Bin Wu

Table of contents

Table S1 2

Table S2. 2

Table S3. 3

Table S4. 4

Supplementary references 5

Table S1: chronic hepatitis C prevalence and population distribution according to age[1–3].

	Estimated Prevalence	Population distribution
<5 years	0.006136158	0.059738982
5-9 years	0.011715877	0.060795336
10-14 years	0.013303903	0.058791425
15-19 years	0.013311699	0.058179866
20-24 years	0.013320962	0.062222495
25-29 years	0.013606482	0.073278142
30-34 years	0.01394847	0.089365186
35-39 years	0.014203694	0.06764186
40-44 years	0.014442049	0.070192224
45-49 years	0.014593455	0.086600454
50-54 years	0.014924339	0.084757947
55-59 years	0.015225713	0.066009365
60-64 years	0.01541528	0.054072897
65-69 years	0.015538108	0.050188477
70-74 years	0.015575276	0.029066774
75-79 years	0.015777422	0.017924958
80+ years	0.016365674	0.018319479
All age	0.0138	1

Table S2: Disease stage distribution according to age.

	<18 years	18-39 years	40-59 years	>=60 years
Fibrosis F0	0.662	0.58391	0.23032	0.076995
Fibrosis F1	0.246	0.27113	0.58391	0.166105
Fibrosis F2	0.071	0.10115	0.27113	0.211645
Fibrosis F3	0.021	0.03055	0.10115	0.22273
Fibrosis F4	0.00001	0.01191	0.03055	0.31904

Table S3: Averted clinical complications in different strategies per 1000 population*.

Strategy	CC	DC	HCC	Excess mortality
Status quo (Reference strategy)	Not applicable	Not applicable	Not applicable	Not applicable
Strategy 1: Screening 18-49 years	273.9(95% UI:186.6 to 354.3)	148(95% UI:98.1 to 198.2)	123.6(95% UI:80.6 to 175.4)	1.76(95% UI:1.2 to 2.27)
Strategy 2: Screening 18-59 years	349.6(95% UI:238.3 to 452.3)	181(95% UI:120.1 to 242.7)	150(95% UI:97.7 to 211.9)	2.3(95% UI:1.59 to 2.96)
Strategy 3: Screening 18-69 years	384.5(95% UI:262.3 to 496.8)	204.8(95% UI:136.1 to 274.1)	167.2(95% UI:109.2 to 235.9)	2.71(95% UI:1.86 to 3.48)
Strategy 4: Screening 18-80 years	398(95% UI:271.3 to 515)	213(95% UI:141.5 to 285.1)	172.8(95% UI:112.9 to 243.4)	2.93(95% UI:2 to 3.78)
Strategy 5: Screening 12-80 years	442.4(95% UI:302.3 to 570.4)	240.1(95% UI:159.6 to 320.5)	196.3(95% UI:128.1 to 276.6)	3.21(95% UI:2.19 to 4.12)
Strategy 6: Screening 3-80 years	495(95% UI:339 to 636)	273.5(95% UI:182.9 to 363.3)	225.5(95% UI:147.4 to 317.7)	3.53(95% UI:2.42 to 4.52)

* Comparing with reference strategy (Status quo).

Table S4: Cost-effectiveness of seven screening strategies in China.

Strategy	cost($)	QALY	LY	ICER($/QALY)*
Status quo (Reference strategy)	19.05 (95% UI: 11.4 to 31.06)	16.506 (95% UI: 12.28 to 1.922)	40.041 (95% UI: 40.019 to 40.062)	NA
Strategy 1: Screening 18-49 years	72.06 (95% UI: 53.92 to 89.42)	16.514 (95% UI: 12.285 to 21.938)	40.072 (95% UI: 40.053 to 40.08)	7868 (95% UI: 3282 to 21655)
Strategy 2: Screening 18-59 years	90.76 (95% UI: 67.59 to 112.22)	16.516 (95% UI: 12.286 to 21.94)	40.075 (95% UI: 40.055 to 40.091)	9687 (95% UI: 3690 to 24288)
Strategy 3: Screening 18-69 years	104.06 (95% UI: 77.11 to 128.7)	16.517 (95% UI: 12.287 to 21.942)	40.077 (95% UI: 40.057 to 40.093)	8191 (95% UI: 3979 to 23056)
Strategy 4: Screening 18-80 years	111.19 (95% UI: 82.29 to 137.69)	16.518 (95% UI: 12.288 to 21.943)	40.077 (95% UI: 40 to 40.094)	7724 (95% UI: 4196 to 23644)
Strategy 5: Screening 12-80 years	118.85 (95% UI: 87.76 to 147.17)	16.519 (95% UI: 12.288 to 21.946)	40.086 (95% UI: 40.066 to 40.103)	9434 (95% UI: 3979 to 23111)
Strategy 6: Screening 3-80 years	128.18 (95% UI: 94.45 to 159.1)	16.521 (95% UI: 12.289 to 21.951)	40.101 (95% UI: 40.078 to 40.11)	9503 (95% UI: 3738 to 22566)

* Comparing with reference strategy (Status quo).

Supplementary references

[1] Assessing the cost-effectiveness of hepatitis C screening strategies in France. Journal of Hepatology 2018;69:785–92. https://doi.org/10.1016/j.jhep.2018.05.027.

[2] Kim H-L, Kim K-A, Choi GH, Jang ES, Ki M, Choi HY, et al. A cost-effectiveness study of universal screening for hepatitis C virus infection in South Korea: A societal perspective. Clin Mol Hepatol 2021;28:91–104. https://doi.org/10.3350/cmh.2021.0236.

[3] Zhou H, Lu Y, Wu B, Che D. Cost-effectiveness of Oral Regimens for Adolescents With Chronic Hepatitis C Virus Infection. Pediatr Infect Dis J 2020;39:e59–65. https://doi.org/10.1097/INF.0000000000002717.
