# Supplementary material for: Universal screening for HCV infection in China: An effectiveness and cost-effectiveness analysis
Source: JHEP Rep. 2024 Jan 11;6(4):101000. doi: 10.1016/j.jhepr.2024.101000 (PMC10933547; doi:10.1016/j.jhepr.2024.101000)
Supplement: Multimedia component 4 [file mmc4.pdf]

Universal screening for HCV infection in China: An effectiveness and cost-effectiveness analysis

Hua Zhou,[1] Mengxia Yan,[2] Datian Che,[1,*] Bin Wu[3,*]

[1]*Department of VIP, Shanghai Children's Hospital, Affiliated with the School of Medicine, Shanghai Jiaotong University, Shanghai, China;* [2]*Department of Pharmacy, Ren Ji Hospital, School of Medicine, Shanghai Jiaotong University, Shanghai, China;* [3]*Clinical Research Institute, Ren Ji Hospital, School of Medicine, Shanghai Jiaotong University, Shanghai, China*

JHEP Reports **2024.** https://doi.org/10.1016/j.jhepr.2024.101000

Background & Aims: Approximately 10 million people live with chronic HCV infection in China, and less than 20% of people with HCV were diagnosed. We aim to determine the cost-effectiveness of one-time HCV screening compared with no screening in the Chinese population from the healthcare system perspective.
Methods: A decision-tree plus Markov model was adopted to project chronic hepatitis C (CHC) prevalence, probability of complications, quality-adjusted life years (QALYs), and costs in the Chinese general population undiagnosed for CHC for different screening strategies. Once CHC was diagnosed, pan-genotypic direct-acting antiviral agent treatment was administered regardless of fibrosis. The population was simulated in a model spanning a lifetime. Input parameters were obtained from published literature. The incremental cost-effectiveness ratio between screening and no screening was estimated. The one-time Chinese gross domestic product per capita in 2021 ($12,558/QALY) was used as the willingness-to-pay threshold.
Results: Universal screening in the population aged 3–80 years led to the lowest probability of complications, which yielded a 62% reduction of excess mortality. Compared with no screening, implementing screening and treatment for HCV in populations aged 3–80 years resulted in the greatest marginal QALYs (15.2 per 1,000 population) with an increase in total costs of $109,136. Calculating the incremental cost-effectiveness ratio yields a value of $9,503/QALY (95% uncertainty interval $3,738–$22,566). The robustness of the model was demonstrated through various sensitivity analyses. If the CHC prevalence was over 0.3%, screening could be cost-effective.
Conclusions: HCV screening for Chinese people aged 3–80 years may be a cost-effective intervention to reduce the disease burden related to HCV infection. This strategy should certainly be implemented.
Impact and implications: This study found that screening Chinese people aged 3–80 years yielded the greatest health benefits and was a cost-effective alternative. The findings indicated that national efforts eliminating HCV should be invested and strengthened in China. The results of this study are important because they provide strong evidence that universal screening can be a cost-effective way to reduce the burden of HCV in China. These findings are important for policymakers, physicians, patients, caregivers, and the public because they promote awareness and inform decision-making for HCV prevention and treatment.
© 2024 The Author(s). Published by Elsevier B.V. on behalf of European Association for the Study of the Liver (EASL). This is an open access article under the CC BY license (http://creativecommons.org/licenses/by/4.0/).

Introduction

HCV remains a major global health issue. The World Health Organization (WHO) estimates that globally, 58 million people had chronic hepatitis C (CHC) infection worldwide in 2019.[1] HCV is one of the leading causes of chronic hepatitis, cirrhosis, and hepatocellular carcinoma (HCC), which resulted in an estimated 0.54 million deaths in 2019, accounting for 0.96% of all deaths worldwide. In 2016, the WHO set its commitment to eliminating primarily HBV and HCV by the year 2030 via a series of therapeutic measures.[2] The coverage targets in 2030 for HBV and HCV are a 90% reduction of incidence and a 65% reduction of mortality related to chronic HBV and HCV infections. In the past two decades, the disease burden related to HBV in China has declined significantly, whereas the disease burden related to HCV has remained stable.[3–5] Therefore, with approximately 10 million chronic carriers, HCV infection is still one of the leading public health challenges in China. Considering that China is not a region with high HCV prevalence in a global perspective, with a highly effective health system for disease control and prevention by the government, HCV eradication is highly feasible.

Currently, proactive screening of HCV in China has primarily targeted high-risk and/or vulnerable populations. However, individuals such as drug users, HIV-infected patients, and men who have sex with men have been neglected because of stigmatization, resulting in a large proportion of new HCV cases being accidentally

Keywords: Hepatitis C screenings; Health outcomes; Economic evaluation.
Received 15 January 2023; received in revised form 20 December 2023; accepted 21 December 2023; available online 11 January 2024
* Corresponding authors. Addresses: Department of VIP, Shanghai Children's Hospital, affiliated with the School of Medicine, Shanghai Jiaotong University, Shanghai, China, 200040. Tel.: +86 21 6247 4880; Fax: +86 21 6247 4880 (D. Che); Medical Decision and Economic Group, Department of Pharmacy, Renji Hospital, affiliated with the School of Medicine, Shanghai Jiaotong University, Shanghai, China, 200127. Tel.: +86 21 6838 3427; Fax: +86 21 6838 342 (B. Wu).
E-mail addresses: cshdoctor@yeah.net (D. Che), scilwsjtu-wb@yahoo.com (B. Wu).

identified among hospitalized patients. In 2021, the Chinese government issued the National Action Plan for Eliminating Hepatitis C as a Public Health Threat (2021–2030) (the "National Plan"), which proposed 15 targets, seven key tasks, and five guaranteeing measures for eliminating viral hepatitis as a public health threat by 2030.[6] However, owing to its large population, low testing rate and poor linkage to care are barriers to eliminating HCV in China.[3–5] Urgent action is needed to scale up screening and treatment efforts aimed at eliminating hepatitis C. At present, the WHO has recommended and promoted the implementation of HCV screening programs. However, universal HCV screening is still absent in the National Health Program. One potential reason is the lack of cost-effective analysis of a universal screening strategy. Thus, the objective of this study was to examine the health and economic benefits of different universal HCV screening strategies in the general Chinese population in comparison with no screening from a healthcare system perspective.

Materials and methods

This analysis was performed according to the Consolidated Health Economic Evaluation Reporting Standards (CHEERS) guideline, which was followed in study assumptions and for reporting the economic study of universal HCV screening in the Chinese population.[7] The Common Rule exempts this study from institutional board review because it does not involve human participants.

Overview of the model

This economic analysis was conducted using a hybrid model that combined a screening decision tree with a lifetime Markov cohort HCV model. The inputs to the model were informed by the literature. In selecting literature for model parameterization, our primary criterion was to give precedence to sources that are derived from systematic reviews, ensuring that the rigor of their methodologies aligns with the standards required for our modeling purposes. The data were gathered through a combination of published literature and consultations with experts in the field. The screening decision tree was adopted to estimate the number of HCV-positive people identified at the population level and the cost of achieving this. The study population consisted of the Chinese general population aged 0–100 years with an unknown diagnosis of CHC. Because direct-acting antiviral agents (DAAs) have been approved in children aged 3–17 years,[8] this study considered not only adults but also children. Therefore, the strategies considered in the analysis included the following (Fig. 1A): the status quo, representing the current approach without any specific screening efforts; and one-time universal screening within specific age ranges, namely 18–49, 18–59, 18–69, and 18–80 years. The upper bound of 80 years includes all individuals who are 80 years old at the time of screening. Currently, the lack of widespread screening programs in China has led to the majority of new HCV cases being incidentally identified among hospitalized patients.

The Markov model incorporated model parameters to account for long-term costs and health outcomes related to HCV screening and treatment (Fig. 1B). According to empirically calibrated models, clinical characteristics, and published literature,[9] the Markov model reflected the natural disease course of HCV infection, which included the following exclusive health states (Fig. 1B): no infection, five METAVIR liver fibrosis states (F0 [no fibrosis] to F4 [cirrhosis]) and decompensated cirrhosis (DC), five METAVIR liver fibrosis states (sustained virological response [SVR] F0–F4) and DC with SVR if the infection was detected and active treatment was administered, HCC, liver transplantation (LT), and death. The cycle length of the Markov model was 1 year, and a lifetime horizon of 100 years was selected according to published literature.[9] In each cycle, the population is either kept in its current state or transitioned to another state, as shown by arrows at the end of each cycle. This is a common simplification in Markov models for chronic diseases, where immediate transitions through multiple health states within a single cycle are not typically modeled owing to their low probability.[9] However, the cumulative effect of possible 1-year transitions over the lifetime horizon encapsulates these low events. The source of the transition probabilities and their details are described in the following paragraph. At the beginning of the model, a hypothetical cohort was assigned to the no infection state and five stages of fibrosis (F0–F4) based on the proportions reported in the literature, which was stratified by population age.[10–12] If patients in stages F0–F2 transitioned to SVR, they were assumed to be cured and not incur recurrence. However, those in stages SVR F3–F4 and SVR DC were allowed to incur histological regression and progress to SVR DC and HCC, albeit at a rate slower than that observed for those in stages F3–F4. Patients with HCV infection in F0–F4 DC can achieve SVR after treatment, whether detected through screening or incidentally. After patients entered into the DC, DC with SVR, HCC, and LT states, they incurred disease-specific mortality. The population without HCV and those in health states other than DC, DC with SVR, HCC, and LT were subject to the Chinese age-specific background mortality provided by the Global Health Estimates.[13]

The health endpoints included the cumulative probability of compensated cirrhosis (CC), DC, and HCC, excess mortality, expected life years (LYs), and quality-adjusted life years (QALYs). Cost and QALYs are annually discounted at 5%.[14] We measured the incremental cost-effectiveness ratio (ICER; US$ per additional QALY gained) of competing strategies compared with that of the status quo (reference strategy). When the estimated ICER was less than $12,558/QALY (the per capita gross domestic product of China in 2021), implicitly accepted as a willingness-to-pay (WTP) threshold in China, the screening strategy would be cost-effective.[14]

Epidemiological and clinical inputs

Model inputs for epidemiology and clinical data were obtained from studies based on the Chinese population to the greatest possible extent (Table 1 and Tables S1 and S2). When no epidemiological studies in China were available, we used data from other countries, especially from East Asia.

In decision trees, the data associated with the prevalence of CHC, treatment effectiveness, and screening were input into the model. Informed by the Global Burden of Disease (GBD) 2019 study,[1] the CHC prevalence was approximately 1.38% across all ages, stratified into 17 intervals by age. The CHC prevalence corresponded to anti-HCV prevalence, taking into account a 30% spontaneous clearance rate.[29] It was assumed that individuals are initially screened for anti-HCV, and if found positive, they undergo HCV RNA confirmatory testing. The sensitivity and specificity of testing anti-HCV were 98.1% and 99.8%, respectively. The acceptance rate of screening was 77% based on a study in East Asia. As indicated in previous studies,[9,11] the overall acceptance rate suggests that people who are willing to be screened for anti-HCV testing are also likely to be willing to

undergo HCV RNA testing because the test would be provided free of charge in the current setting. We also assumed that only one-time screening for HCV infection was provided to the target population. In the absence of a formal screening program, we assumed an incidental detection rate of 18% per year for HCV based on the literature.[25,26] This rate reflects the proportion of individuals with previously undiagnosed active CHC who are identified each year through non-targeted medical interactions. When patients were diagnosed with CHC, pan-genotypic DAAs would be administered. The proportions of accepting treatment were 62% and 88% in the scenarios of passive and active screening,[30] respectively. Once CHC was confirmed, patients

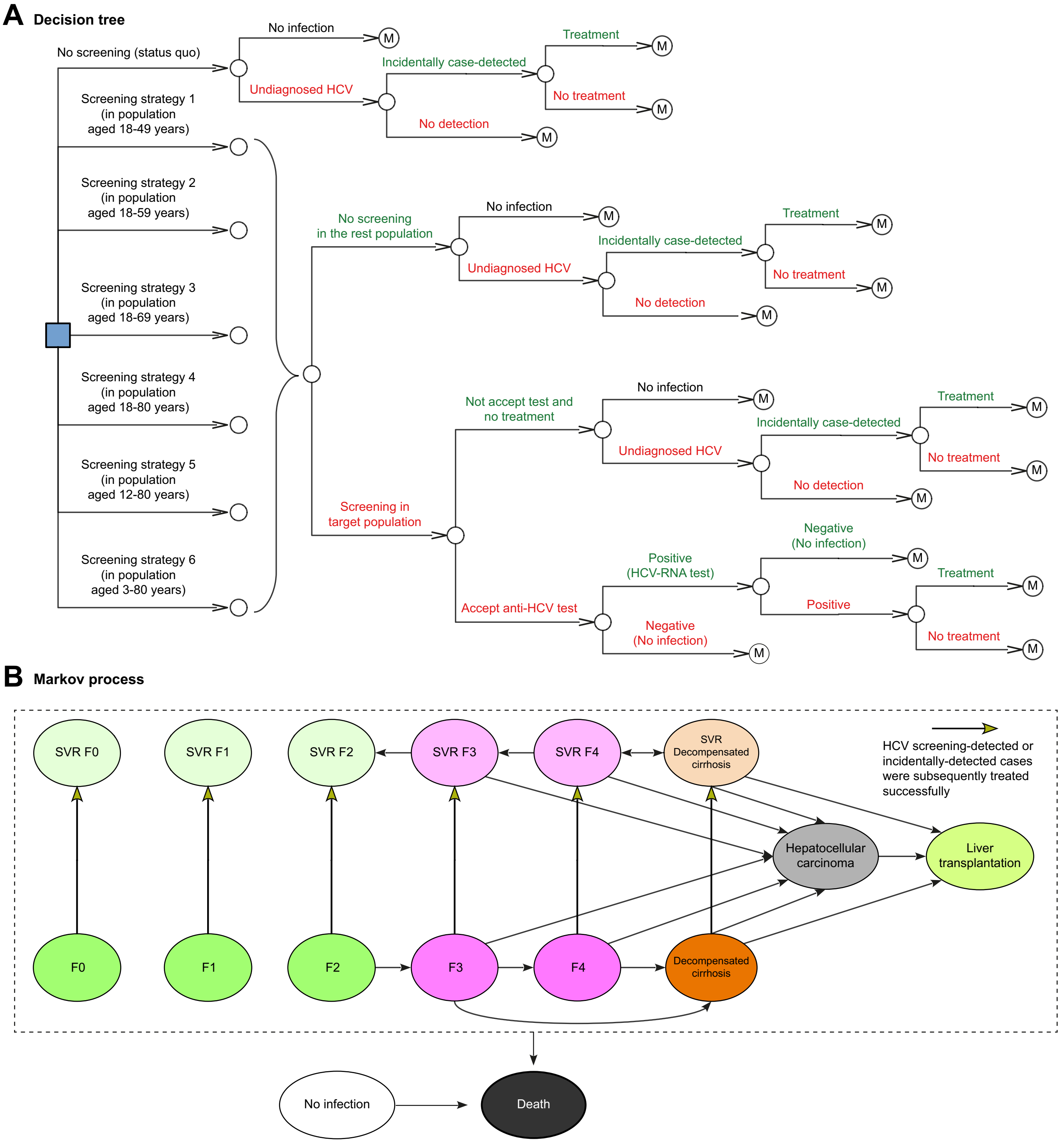

Fig. 1. Model structure. (A) The decision tree illustrates seven screening scenarios: "no screening" with incidentally detected cases and six "one-off" positively screened strategies, all followed by a treatment approach. The intervention outcomes, regardless of whether patients are detected through screening or incidentally, are subsequently incorporated into the Markov model through distinct transition states. (B) In each model cycle, patients may transition between health states based on a specified transition probability. SVR, sustained virological response.

Table 1. Model inputs.

Parameters	Expected values (ranges*)	Distributions	References
**Clinical data**			
Transition probabilities			
F0 → F1 per year	0.117 (0.104–0.13)	Beta (274.8, 2073.6)	Thein *et al.*[15]
F1 → F2 per year	0.085 (0.075–0.096)	Beta (230.4, 2479.7)	Thein *et al.*[15]
F2 → F3 per year	0.121 (0.109–0.133)	Beta (343.3, 2494.1)	Thein *et al.*[15]
F3 → F4 per year	0.116 (0.104–0.129)	Beta (292.5, 2228.7)	Thein *et al.*[15]
F3 → DC per year	0.012 (0.009–0.015)	Beta (15.8, 1301.5)	Chahal *et al.*[16]
F4 → DC per year	0.039 (0.029–0.049)	Beta (15.4, 378.9)	Chahal *et al.*[16]
F3 → HCC per year	0.011 (0.008–0.014)	Beta (15.8, 1422.7)	Chahal *et al.*[16]
F4 → HCC per year	0.024 (0.018–0.03)	Beta (15.6, 635.1)	Chahal *et al.*[16]
SVR F3 →SVR F2 per year	0.267 (0.2–0.334)	Beta (45.1, 123.7)	Maylin *et al.*[17]
SVR F4 →SVR F3 per year	0.076 (0.057–0.095)	Beta (56.8, 690.5)	D'Ambrosio and Aghemo[18]
SVR DC → SVR F4 per year	0.076 (0.057–0.095)	Beta (56.8, 690.5)	Assumed[†]
SVR F4 → DC per year	0.003 (0.002–0.004)	Beta (16, 5301.4)	Zhou *et al.*[12]
SVR F3 → HCC per year	0.003 (0.002–0.003)	Beta (20.6, 6845.8)	Zhou *et al.*[12]
SVR F4 → HCC per year	0.006 (0.005–0.008)	Beta (15.9, 2634.8)	Zhou *et al.*[12]
DC → HCC per year	0.014 (0.01–0.083)	Beta (0.6, 39.2)	Zhou *et al.*[12]
SVR DC → HCC per year	0.003 (0.003–0.004)	Beta (12.7, 4226.2)	Fattovich *et al.*,[19] Morgan *et al.*[20]
DC/SVR DC → LT per year	0.0003 (0.0002–0.0011)	Beta (1.9, 6023.7)	Zhou *et al.*[12]
DC → Death per year	0.052 (0.032–0.084)	Beta (14.5, 265.4)	Zhou *et al.*[12]
SVR DC → Death per year	0.042 (0.032–0.053)	Beta (15.3, 349.6)	Saab *et al.*[21]
HCC → LT per year	0.0005 (0–0.0024)	Beta (0.6, 1212.5)	Zhou *et al.*[12]
HCC → Death per year	0.368 (0.36–0.375)	Beta (5813, 9992)	Zhou *et al.*[12]
LT → Death in the first year	0.2187 (0.164–0.2734)	Beta (12.5, 44.7)	Zhou *et al.*[12]
LT → Death in the subsequent year	0.0668 (0.0501–0.0835)	Beta (14.9, 208.5)	Zhou *et al.*[12]
HR of fibrosis progression between Asian and Western	1.28 (1.02–1.61)	Normal (1.28, 0.151)	Le *et al.*[22]
Probability of SVR			
Pan-genotypic DAA in patients with F0–F4	0.96 (0.95–0.97)	Beta (1416.2, 59)	Xie *et al.*[23]
Pan-genotypic DAA in patients with DC	0.923 (0.83–0.975)	Beta (47.9, 4)	Tada *et al.*[24]
Anti-HCV test			
Sensitivity	0.981 (0.926–0.997)	Beta (55.7, 1.1)	Kim *et al.*[11]
Specificity	0.998 (0.992–0.999)	Beta (624.7, 1.3)	Kim *et al.*[11]
Prevalence of CHC in China stratified by age[‡]	Table S1	Beta	GBD 2019[1]
Distribution of fibrosis stage stratified by age[§]	Table S2	Beta	Deuffic-Burban *et al.*,[10] Zhou *et al.*[12]
Acceptability of screening	0.77 (0.5–1)	Beta (8.3, 2.4)	Kim *et al.*[11]
Detection rate without screening	0.18 (0.12–0.34)	Beta (8.4, 38.4)	Li *et al.*,[25] Rein *et al.*[26]
Acceptability of treatment after passive diagnosis	0.62 (0.39–0.734)	Beta (19, 11.6)	Lin *et al.*,[3] Song *et al.*[4]
Acceptability of treatment after active screening and CHC diagnosis	0.88 (0.85–0.93)	Beta (201.8, 27.6)	Liu *et al.*[5]
**Cost data**			
Anti-HCV test per unit	3.14 (2.35–3.92)	Gamma (784.88, 0.004)	Heffernan *et al.*[27]
HCV-RNA test per unit	43.95 (32.97–54.94)	Gamma (15.1, 2.91)	Heffernan *et al.*[27]
Pan genotypic DAA per patient	1,781 (1,530–2,032)	Gamma (24735, 0.072)	Chinese list prices
Annually managing F0–F3 HCV disease	924 (625–1,223)	Gamma (5601, 0.165)	Zhou *et al.*[12]
Annually managing F4 HCV disease	2,630 (932–4,328)	Gamma (7995, 0.329)	Zhou *et al.*[12]
Annually managing DC disease	5,858 (3,559–8,157)	Gamma (29290, 0.2)	Zhou *et al.*[12]
Annually managing HCC disease	12,365 (8,892–15,839)	Gamma (86469, 0.143)	Zhou *et al.*[12]
Annually managing LT in the first year	53,643 (38,760–77,519)	Gamma (291540, 0.184)	Zhou *et al.*[12]
Annually managing LT in the subsequent year	8,527 (7,751–9,530)	Gamma (160889, 0.053)	Zhou *et al.*[12]
Relative costs in post SVR F3–F4	0.709 (0.592–0.855)	Normal (0.709, 0.01)	Zhou *et al.*[12]
**Utility data**			
F0–F1	0.853 (0.765–0.95)	Beta (48, 8.3)	Zhou *et al.*,[12] Saeed *et al.*[28]
F2–F3	0.853 (0.765–0.95)	Beta (48, 8.3)	Zhou *et al.*,[12] Saeed *et al.*[28]
F4	0.773 (0.68–0.876)	Beta (54.3, 15.9)	Zhou *et al.*,[12] Saeed *et al.*[28]
SVR F0–F1	0.888 (0.8–0.985)	Beta (39.7, 5)	Zhou *et al.*,[12] Saeed *et al.*[28]
SVR F2	0.888 (0.8–0.985)	Beta (39.7, 5)	Zhou *et al.*,[12]Saeed *et al.*[28]
SVR F3	0.888 (0.8–0.985)	Beta (39.7, 5)	Zhou *et al.*,[12] Saeed *et al.*[28]
SVR F4	0.888 (0.8–0.985)	Beta (39.7, 5)	Zhou *et al.*,[12] Saeed *et al.*[28]
DC	0.704 (0.603–0.816)	Beta (65.9, 27.7)	Zhou *et al.*,[12] Saeed *et al.*[28]
SVR DC	0.784 (0.688–0.89)	Beta (59.6, 16.4)	Zhou *et al.*,[12] Saeed *et al.*[28]
HCC	0.765 (0.652–0.885)	Beta (46.6, 14.3)	Zhou *et al.*,[12] Saeed *et al.*[28]
LT in the first year	0.663 (0.563–0.8)	Beta (66.5, 33.8)	Zhou *et al.*,[12] Saeed *et al.*[28]
LT in the subsequent year	0.759 (0.657–0.87)	Beta (39.3, 12.5)	Zhou *et al.*,[12] Saeed *et al.*[28]

* Ranges were determined either from the published variance, when available, or by applying a ±25% adjustment to their base-case values when no variance was reported, except for the parameter "Acceptability of screening," for which a broad range was assumed to assess its potential impact.
† This probability was assumed to be similar to SVR F4 →SVR F3.
‡ Prevalence of CHC in China was stratified by the following 17 age groups: <5, 5–9, 10–14, 15–19, 20–24, 25–29, 30–34, 35–39, 40–44, 45–49, 50–54, 55–59, 60–64, 65–69, 70–74, 75–79, and 80+ years.
§ Distribution of fibrosis stage was stratified by the following age groups: <18, 18–39, 40–59, and 60+ years. CC, compensated cirrhosis; CHC, chronic hepatitis C; DAA, direct-acting antiviral agent; DC, decompensated cirrhosis; F0–F4, METAVIR liver fibrosis scores; HCC, hepatocellular carcinoma; HR, hazard ratio; LT, liver transplantation; SVR, sustained virological response.

were allocated pan-genotypic DAA treatment. The SVR rate of pan-genotypic DAA treatment was collected from real-world evidence, with rates of 96% (95% CI 95–97%) and 92.3% (95% CI 83.0–97.5%) in patients with F0–F4 fibrosis and DC,[23,24] respectively. Owing to the relatively highly favorable safety profiles and short treatment courses related to DAA therapies, this analysis did not consider impacts associated with retreatment, treatment-related adverse events, utility decrements, and discontinuation.

In the Markov model, the inputs included transition probability and fibrosis distribution.[15–20] Because of mild or no symptoms in patients diagnosed through screening, no symptomatic cases of DC were assumed at the time points of model entry. Probabilities related to the transition between Markov states were determined as the preferred values by expert opinion through literature reviews or from a previously published model. Re-infection was excluded from this analysis because there were limited data and it was considered to be very rare in China.

Costs and utility inputs

This analysis adopted the healthcare perspective of China, which considered only direct medical costs, including screening and diagnosis, antiviral therapy, and the management of complications associated with HCV (Table 1). All Chinese costs were reported in 2021 US dollars (US$1 = 6.45 CNY). In China, the costs for the HCV antibody test and HCV RNA quantitative test per unit were $3.14 and $43.95, respectively.[27] The costs of pan-genotypic DAA treatment per patient were $1,781 per 12-week treatment course, which were sourced from Chinese list prices. Within the natural history model, the annual direct medical costs of managing patients with a METAVIR score of F0–F4, DC, HCC, and LT were taken from our previous economic study.[12] As previously studied,[9,11] patients with SVR in stages F0–F2 were assumed to incur no direct medical costs, as these patients are considered cured and no longer require ongoing treatment or are expected to develop any other medical complications. Patients with SVR in stages F3–F4 used fewer health resources than those without SVR. The relative ratio of the costs in stages F3–F4 with SVR *vs.* without SVR was 0.709.[12] Patients without HCV did not incur any medical costs.

Utility scores were assigned for each health state, and they were gathered from the published literature (Table 1).[12,28]

Analysis

Deterministic and probabilistic sensitivity analyses (PSA) were conducted to explore the uncertainty of the model variables and applied assumptions. In the PSA, 10,000 Monte Carlo simulations were run by inputting parameters sampled from their statistical distributions as presented in Table 1 (*i.e.* gamma distribution for costs; normal distribution for log risk ratios (RRs) and health resource utilization; and beta distribution for utilities, probabilities, and proportions). The results of the PSA were presented as the mean value with 95% uncertainty intervals (UIs), which were estimated by setting the lower and upper bounds as the 2.5th and 97.5th percentiles, respectively. A 95% UI not containing zero was considered to indicate statistically significance. Using the results of the PSA, the cost-effectiveness acceptability curve (CEAC) was constructed to present uncertainty surrounding ICERs. The CEAC indicates the probability that the strategy is cost-effective compared with the alternatives at different WTP thresholds.[31] In the deterministic sensitivity analysis, the gaps in the ICERs of a variable between lower and upper boundaries (Table 1 showed the range) were calculated, and the results are presented as a tornado graph. Model development and all analyses were performed using R version 4.1.1 (R Foundation for Statistical Computing, Vienna, Austria), incorporating standard/published R functions from widely used packages, such as heemod.

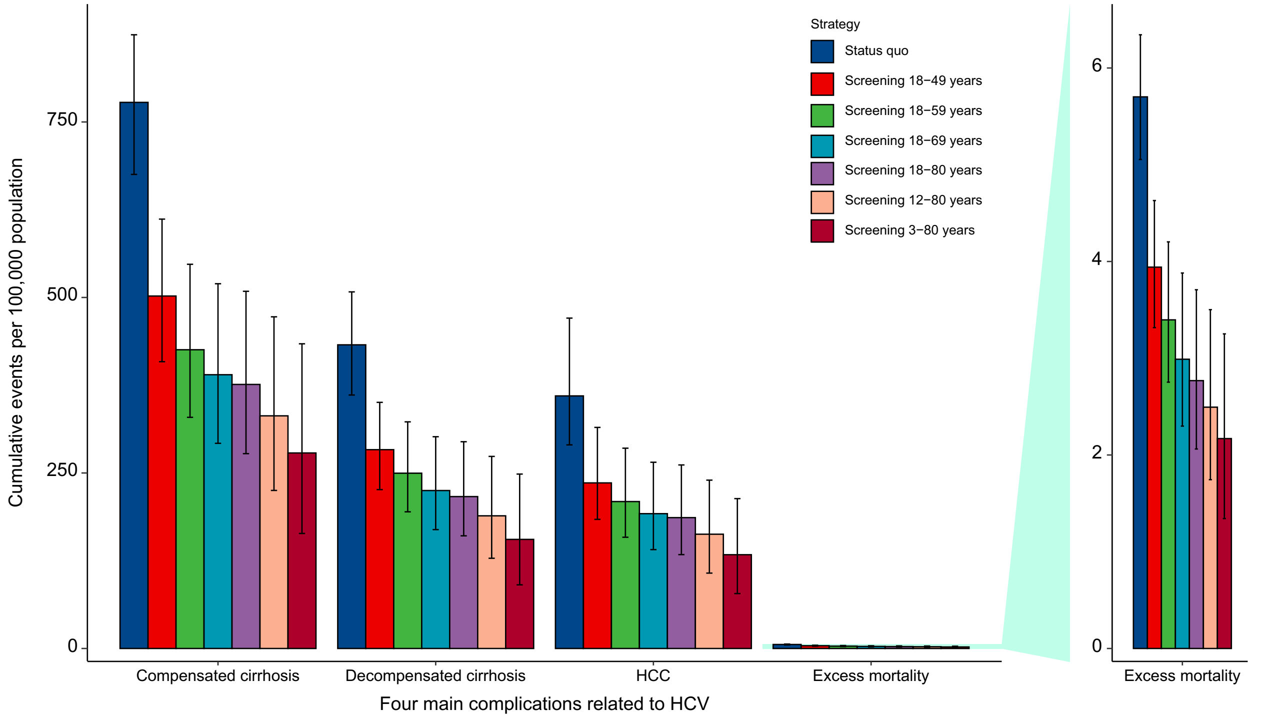

Fig. 2. Four main complications related to HCV in the status quo and six screening strategies. HCC, hepatocellular carcinoma.

Results

Health-related outcomes

When considering screening implementation to populations aged 18–49, 18–59,18–69, 18–80, 12–80, and 3–80 years, undiagnosed CHC prevalence among the whole general population decreased to 0.71% (95% UI 0.58–0.87%), 0.57% (95% UI 0.43–0.75%), 0.47% (95% UI 0.31–0.68%), 0.41% (95% UI 0.24–0.64%), and 0.35% (95% UI 0.18–0.59%), respectively. These values were substantially lower than that of the status quo (1.12% [95% UI 0.97–1.23%]).

In the status quo, the excess mortality related to HCV infection was approximately 5.70 (95% UI 5.05–6.34) per 100,000 populations (Fig. 2), which was comparable with the reported GBD result (5.49 [95% UI 4.53–6.48]).[1] Compared with those in the status quo, the numbers of averted CCs, DCs, and HCCs over a lifetime were gradually augmented by expanding the target population, which was from 273.9 (95% UI 186.6–354.3) to 495 (95% UI 339–636), from 148 (95% UI 98.1–198.2) to 273.5 (95% UI 182.9–363.3), and from 123.6 (95% UI 80.6–175.4) to 225.5 (95% UI 147.4–317.7) per 100,000 populations from strategy 1 to strategy 6, respectively (Fig. 2 and Table S3). Moreover, HCV-related deaths per 100,000 populations over a lifetime were estimated to be reduced from 1.76 (95% UI 1.2–2.27) in strategy 1 to 3.53 (95% UI 2.42–4.52) in strategy 6.

Cost-effectiveness analysis

Fig. 3 and Table S4 present the findings of the economic outcomes using PSA for various HCV screening strategies. Compared with those in the status quo, the incremental costs per 1,000 population were also gradually augmented by expanding the target population, which ranged from $53,014 (95% UI $36,829–$68,375) in screening strategy 1 to $10,9136 (95% UI $76,306–$139,687) in screening strategy 6, and the incremental QALYs per person were from 8.20 (95% UI 2.30–16.80) in screening strategy 1 to 15.20 (95% UI 4.5–30.4) in screening strategy 6. Fig. 3A also demonstrated the incremental costs and QALYs associated with each screening strategy relative to the other screening strategies. In comparing each strategy, the screening strategy with a larger

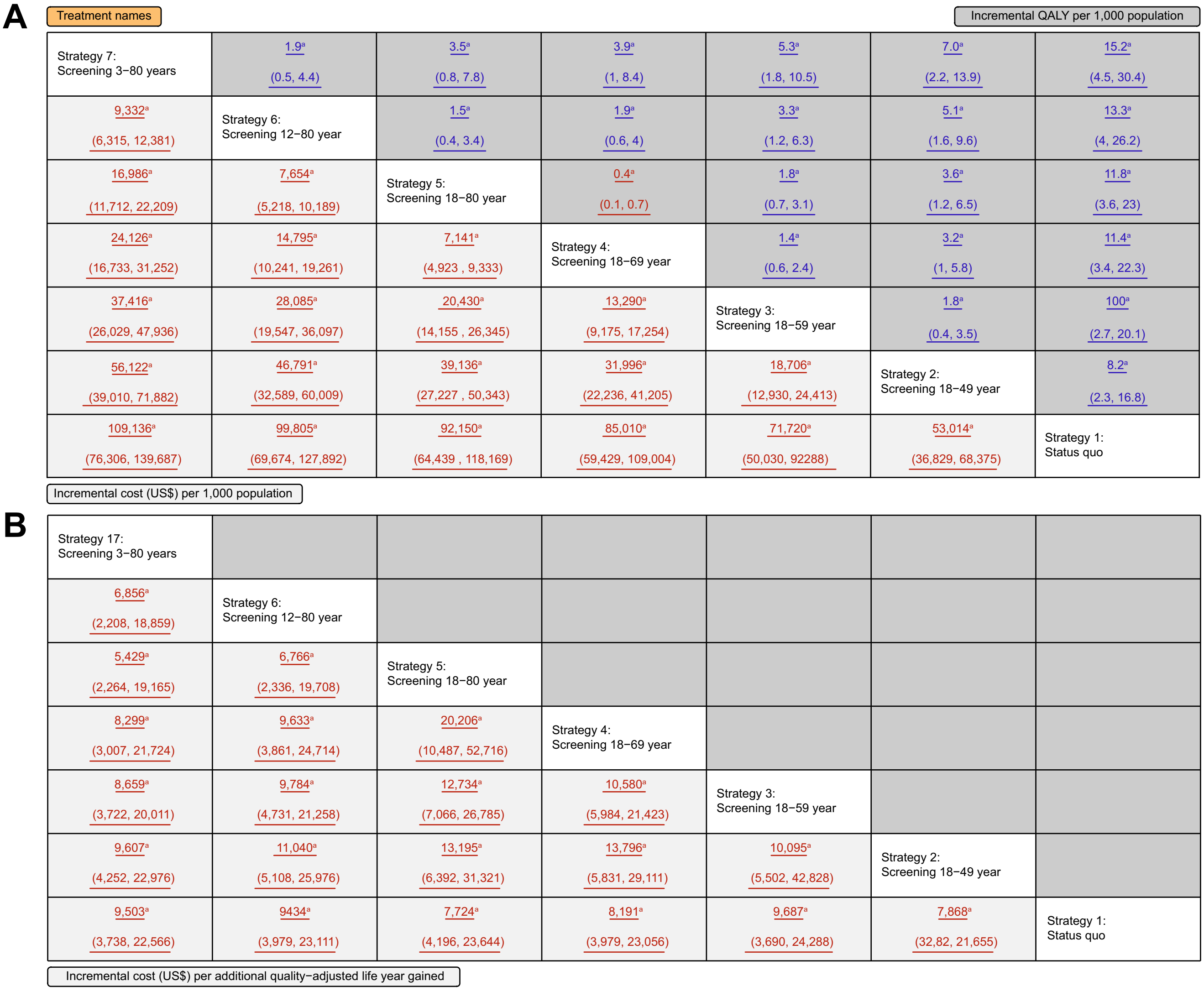

Fig. 3. Comparing the cost and QALYs and increment cost per additional QALY and LY gained among seven strategies. LY, life year; QALY, quality-adjusted life year.

target population exhibited a corresponding increase in both incremental costs and QALYs. Among six active screening strategies, significant differences in cost and QALY were observed when compared with each other. The notable ICERs of screening strategies *vs.* the status quo ranged from $7,724 (95% UI $4,196–$23,644) in strategy 4 to $9,687 (95% UI $3,690–$24,288) in strategy 2 (Fig. 3B). The ICERs of strategy 6 (screening 3–80 years) *vs.* the rest of the screening strategies were likely to be below the threshold of $12,588/QALY.

Sensitivity analysis

The cost-effectiveness acceptability curves showed that compared with the other six strategies, strategy 6 (screening 3–80 years) yielded an 84% probability of cost-effectiveness at the threshold of $12,588/QALY (Fig. 4).

The tornado diagrams showed the comparison between strategy 6 (screening 3–80 years) and the reference strategy because strategy 6 achieved the greatest health outcomes and highest probabilities of cost-effectiveness (Fig. 5). The deterministic sensitivity analyses revealed that the results of the model were more sensitive to the discount rate and sensitivity of the anti-HCV test, whose upper and lower values could lead the ICERs of strategy 6 *vs.* the reference strategy to be higher than the threshold ($12,588/QALY), respectively. The rest of the variables have moderate or small effects and did not lead the ICER to exceed the threshold.

To explore the potential impact of HCV prevalence, further one-way sensitivity analyses were performed by using a wider range. The ICERs of all six screening strategies *vs.* the status quo become more favorable with the higher prevalence. When the prevalence of HCV was below 0.3%, the ICERs for some screening strategies might surpass the threshold (Fig. 6).

Discussion

This study reported the health and economic outcomes of six HCV screening strategies compared with no screening for HCV in China. First, in the status quo, the adverse complications of CHC led to an increasing public health burden, which was associated with the lowest life expectancy and QALYs. Excess mortality from HCV is forecasted to be less than five per 100,000 population. Second, the one-time universal screening of population with specific age improved health outcomes by decreasing the cumulative probability of adverse complications and excess mortality, which were

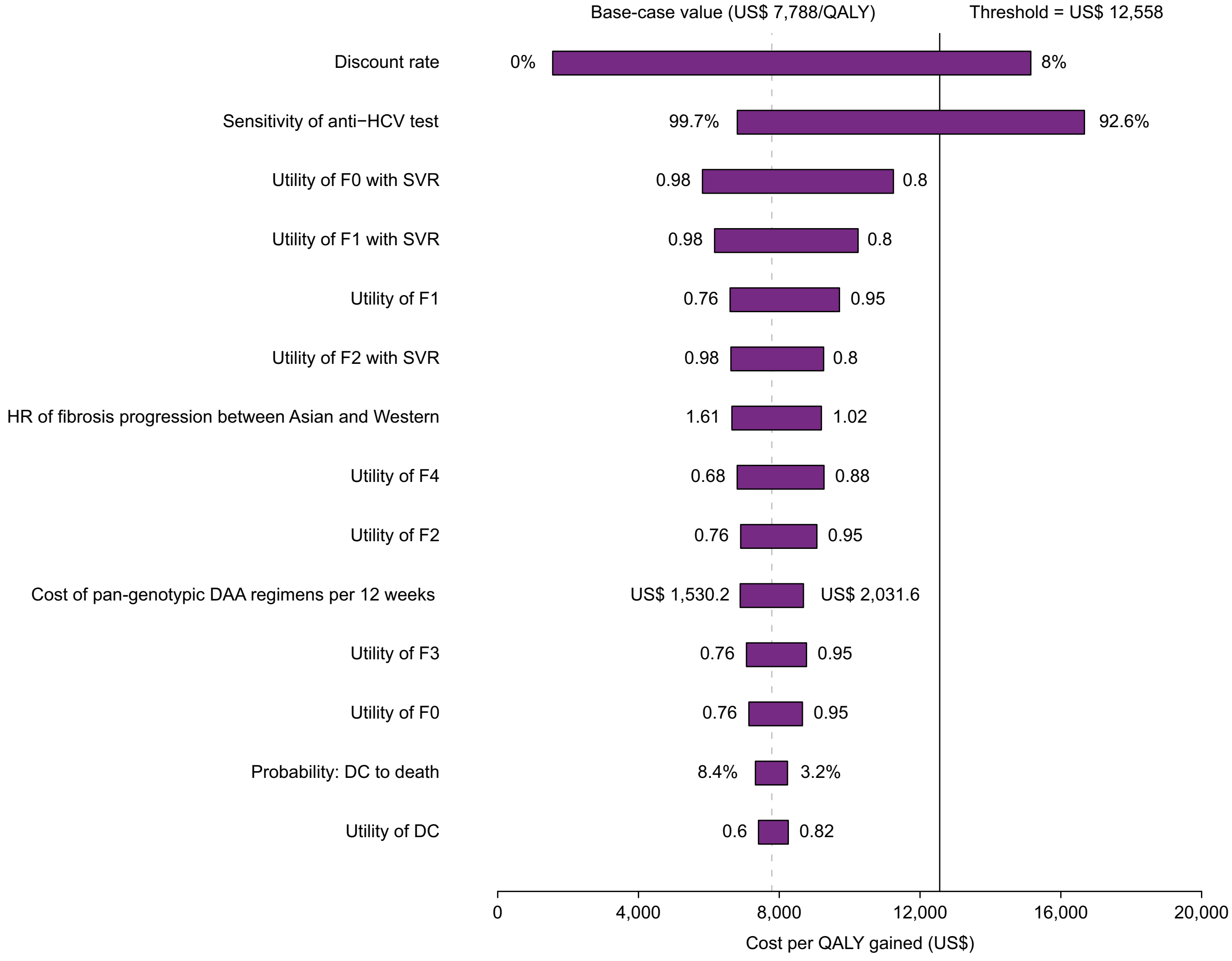

Fig. 4. Cost-effectiveness acceptability curve for seven strategies. DAA, direct-acting antiviral agent; DC, decompensated cirrhosis; HR, hazard ratio; QALY, quality-adjusted life year; SVR, sustained virological response.

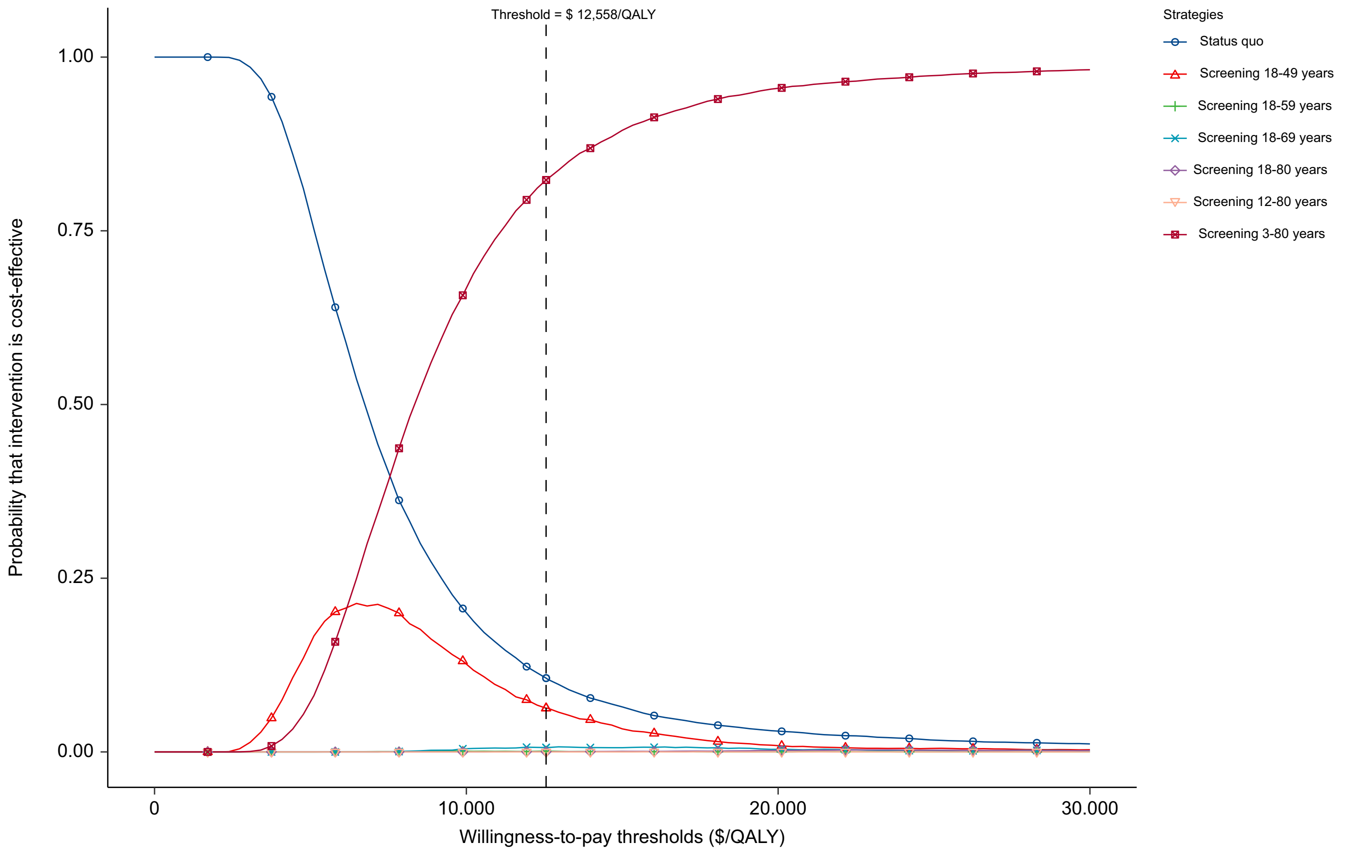

Fig. 5. Tornado diagrams showing the lower and upper values of each parameter in the ICER of strategy 6 (screening 3–80 years) *vs.* the status quo (reference strategy) in the Chinese population. ICER, incremental cost-effectiveness ratio; QALY, quality-adjusted life year.

associated with higher life expectancy and QALYs; the addition of all populations aged 3–80 years to the status quo achieved the greatest health outcomes, including the 62% reduction of excess mortality. Third, the implementation of screening was associated with a higher cost as a result of the augmented cost of screening and DAA treatment. However, the ICERs of all six screening strategies against the status quo was far less than $12,588, which is the implicit WTP threshold in China, based on the country's gross domestic product per capita in 2021. When we considered the health outcomes, universal screening in all populations aged 3–80 years might be a preferred cost-effective option for reducing the disease burden. The early intervention may help prevent disease progression, which can have a significant impact on a child's overall health trajectory into adulthood. This includes improved long-term outcomes and reduced healthcare costs associated with complications. The key findings of this study might inform Chinese policymakers of the health economic value of screening for HCV.

Generally, screening drug users, birth cohorts, high-risk populations, and the general population appear to be a good value for money if a cost per QALY of $40,130 is used as the threshold for reasonable value.[9] Our overall findings were coherent with these previous reports that indicated the cost-effectiveness of universal HCV screening compared with no screening in various countries. In one economic evaluation study, universal screening was found to be cost-effective because the estimated ICER was approximately from $1,003 in people who inject drugs to $11,756 in pregnant women per disability-adjusted life year in the specific case of Yunnan province, which has a large population of people who inject drugs in China.[27] However, there are large gaps between targets and current progress, including insufficiently testing high-risk populations as a result of stigma and discrimination in China. Therefore, screening the general population might be a more rational strategy instead of screening high-risk populations, which is important to fully promote and implement the national hepatitis C elimination action plan. In addition to the lower cost of pan-genotypic DAA treatment in China (<$2,000 per patient), increased treatment acceptance after screening should be improved to save overall costs. Our one-way sensitivity analyses found that the increased probabilities of screening and treatment led to a slightly lower ICER than that seen in the base-case analysis.

The CHC prevalence from the GBD 2019 study was adopted in the base-case analysis (all ages: 1.38%), and sensitivity analyses showed that the estimated ICERs of all universal screening strategies could be kept under the threshold even if the prevalence was lowered by 0.3%. However, it should be noted that the low CHC prevalence could lead the universal screening strategies to be not cost-effective because the ICERs would exceed the threshold. In the scenario of low CHC prevalence, the expenses increased for HCV screening tests and could not be offset by early diagnosis and treatment of CHC. In a society with low CHC prevalence, target screening in high-risk populations, such as routine screening for HCV infection in hospitalized patients,

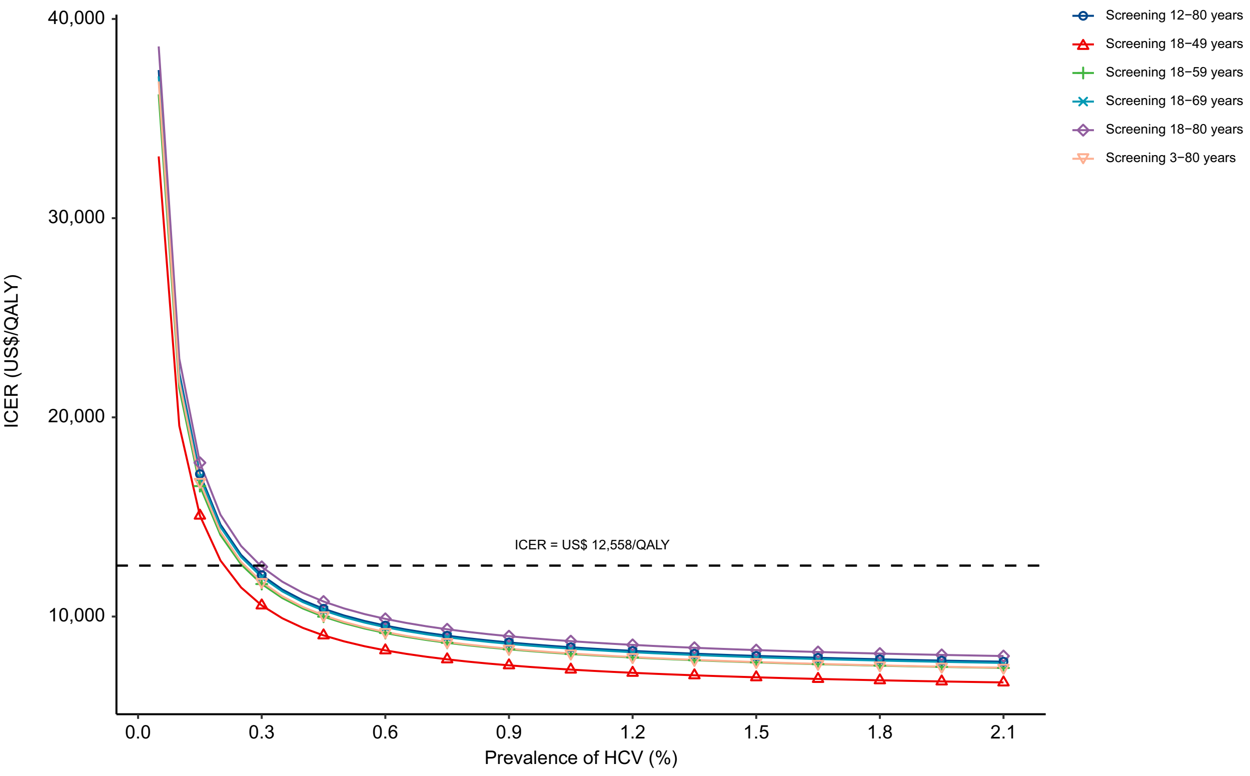

Fig. 6. The impacts of HCV prevalence on the ICERs of six screening strategies *vs.* status quo. ICER, incremental cost-effectiveness ratio; QALY, quality-adjusted life year.

might be an alternative strategy. This finding was also similar to previous economic evaluations that demonstrated screening populations with a higher prevalence of HCV (*i.e.* drug users) generally resulted in better value for money.[9] In addition, the robustness of model outcomes for universal screening was examined through various sensitivity analyses.

Few variables affected the model outcomes, except the discount rate and the performance of the anti-HCV test. A higher discount rate could result in less favorable ICERs owing to the effect of discounting the cost-effectiveness of screening strategies because costs associated with screening the treatment occur early and most economic and health benefits are realized in the future. However, a previous study reported that constant discount rates strongly devalue the long-term health benefits of prevention, which is unlikely to reflect societal preferences,[32] especially among young people. Future studies are warranted to examine the rationale behind using age-specific discount rates in economic evaluations. In cases in which the key clinical parameter is related to the sensitivity of the anti-HCV test, the ICERs of the screening strategies *vs.* the status quo could also become less favorable because adjusting this parameter weakened the effectiveness of finding a missing patient. A recent systematic review found that the pooled sensitivity and specificity of new point-of-care HCV RNA assays were 99% (95% CI 98–99%) and 99% (95% CI 99–100%), respectively.[33] Because the higher sensitivity of test could lead the screening strategy to be more cost-effective, adopting the new point-of-care assays should be considered, particularly if their cost is comparable with that of the current test. It is also important to mention that this study assumes a two-step screening process involving antibody and RNA tests. Although this process holds potential for enhancing cost-effectiveness by optimizing resource allocation, there is a possibility that certain patients may go unrecognized if their first test yields a negative result. In addition, some individuals may choose not to undergo additional tests because of personal preferences or convenience factors.

This study had some limitations. First, it did not adopt societal and whole-of-disease perspectives, as this approach is not routinely recommended in the Chinese setting. Societal perspectives generally yield more favorable outcomes than healthcare system perspectives, as they account for indirect costs that are not captured by healthcare systems.[11] However, the possibility of decreased (or increased) risks for other diseases after HCV treatment could lead to overestimation or underestimation of the conclusions. Second, the prevalence of HCV infection in China was based on the GBD data. However, the GBD estimations were reconstructed through an algorithm based on a large number of sources with different qualities, which, to some degree, could deviate from the actual data. Third, potential HCV reinfection was not considered in this model because the reinfection in the general population was low. However, HCV reinfection rates are high among some high-risk populations (11%).[34] This assumption might overestimate the benefits of screening strategies. Fourth, the current analysis did not account for the potential impact of age-related differences in the disease progression and acceptability of testing and treatment owing to the limited availability of robust evidence.[35] It is important to acknowledge that when these inputs occur at varying rates within specific age groups, there is a possibility of overestimating or underestimating the economic outcomes associated with

screening. Fifth, although the present study did not conduct an independent systematic review to curate our model's parameters, prioritizing sources from existing systematic reviews has ensured a high level of scrutiny and synthesis, thereby supporting the credibility of our input data. Finally, we used a mathematical model with inputs from multiple sources, including some single studies, which may introduce some uncertainty. Future studies need to expand the dataset to improve the accuracy of the parameters.

At present, the task of greatest priority in China is improving the screening system to identify the infected population as soon as possible. Our study demonstrated that the one-time universal HCV screening of all individuals aged 3–80 years is the most effective and cost-effective strategy compared with no screening. As pan-genotypic DAAs are covered by the reimbursement list of medical items in China, incorporating universal HCV screening into the national program can help achieve the goal of HCV elimination in the country.[6]

Abbreviations

CC, compensated cirrhosis; CEAC, cost-effectiveness acceptability curve; CHC, chronic hepatitis C; DC, decompensated cirrhosis; DAA, direct-acting antiviral agent; HR, hazard ratio; HCC, hepatocellular carcinoma; ICER, incremental cost-effectiveness ratio; LT, liver transplantation; LY, life year; PSA, probabilistic sensitivity analysis; QALY, quality-adjusted life year; SVR, sustained virological response; UI, uncertainty interval; WHO, World Health Organization; WTP, willingness to pay.

Financial support

This study was supported by a grant from the National Natural Science Foundation of China (72074142). The funders were not involved in the collection, analysis, or interpretation of data or in the writing or submitting of this report.

Conflicts of interest

The authors declare no conflicts of interest that pertain to this work.

Please refer to the accompanying ICMJE disclosure forms for further details.

Authors' contributions

Conceived the study and are the guarantors: BW, DC. Drafted the manuscript: BW. Collected and analysed the data: MY, DC. Revised and approved the final version of the manuscript: BW. Participated in the data preparation and provided important comments on the manuscript: MY, DC. Read and approved the final manuscript and are accountable for all aspects of the work, including accuracy and integrity: all authors.

Data availability statement

All data were derived from public reports and database. Information on how to access the data can be found in the references cited in this paper.

Supplementary data

Supplementary data to this article can be found online at https://doi.org/10.1016/j.jhepr.2024.101000.

References

[1] GBD 2019 Diseases and Injuries Collaborators. Global burden of 369 diseases and injuries in 204 countries and territories, 1990-2019: a systematic analysis for the Global Burden of Disease Study 2019. Lancet 2020;396:1204–1222.
[2] Cox AL, El-Sayed MH, Kao J-H, et al. Progress towards elimination goals for viral hepatitis. Nat Rev Gastroenterol Hepatol 2020;17:533–542.
[3] Lin C, Clark R, Tu P, et al. The disconnect in hepatitis screening: participation rates, awareness of infection status, and treatment-seeking behavior. J Glob Health 2019;9:010426.
[4] Song Y, Li Y, Cheng H, et al. Eliminate hepatitis C as a public health threat: a narrative review of strategies, gaps, and opportunities for China. Infect Dis Ther 2022;11:1427–1442.
[5] Liu Y, Zou X, Chen W, et al. Hepatitis C virus treatment status and barriers among patients in methadone maintenance treatment clinics in Guangdong Province, China: a cross-sectional, observational study. Int J Environ Res Public Health 2019;16:4436.
[6] Li J, Pang L, Liu Z. Interpretation of the national action plan for eliminating hepatitis C as a public health threat (2021–2030). China CDC Wkly 2022;4:627–630.
[7] Husereau D, Drummond M, Augustovski F, et al. Consolidated health economic evaluation reporting standards (CHEERS) 2022 explanation and elaboration: a report of the ISPOR CHEERS II good practices task force. Value Health 2022;25:10–31.
[8] Rubino C, Trapani S, Indolfi G. Sofosbuvir/velpatasvir for the treatment of hepatitis C in pediatric patients. Expert Rev Gastroenterol Hepatol 2021;15:1097–1105.
[9] Coward S, Leggett L, Kaplan GG, et al. Cost-effectiveness of screening for hepatitis C virus: a systematic review of economic evaluations. BMJ Open 2016;6:e011821.
[10] Deuffic-Burban S, Huneau A, Verleene A, et al. Assessing the cost-effectiveness of hepatitis C screening strategies in France. J Hepatol 2018;69:785–792.
[11] Kim H-L, Kim K-A, Choi GH, et al. A cost-effectiveness study of universal screening for hepatitis C virus infection in South Korea: a societal perspective. Clin Mol Hepatol 2021;28:91–104.
[12] Zhou H, Lu Y, Wu B, et al. Cost-effectiveness of oral regimens for adolescents with chronic hepatitis C virus infection. Pediatr Infect Dis J 2020;39:e59–e65.
[13] WHO | Life tables n.d. https://www.who.int/gho/mortality_burden_disease/life_tables/life_tables/en/ Accessed 22 November 2023.
[14] Yue X, Li Y, Wu J, et al. Current development and practice of pharmacoeconomic evaluation guidelines for universal health coverage in China. Value Health Reg Issues 2021;24:1–5.
[15] Thein H-H, Yi Q, Dore GJ, et al. Estimation of stage-specific fibrosis progression rates in chronic hepatitis C virus infection: a meta-analysis and meta-regression. Hepatology 2008;48:418–431.
[16] Chahal HS, Marseille EA, Tice JA, et al. Cost-effectiveness of early treatment of hepatitis C virus genotype 1 by stage of liver fibrosis in a US treatment-naive population. JAMA Intern Med 2016;176:65–73.
[17] Maylin S, Martinot-Peignoux M, Moucari R, et al. Eradication of hepatitis C virus in patients successfully treated for chronic hepatitis C. Gastroenterology 2008;135:821–829.
[18] D'Ambrosio R, Aghemo A. Treatment of patients with HCV related cirrhosis: many rewards with very few risks. Hepat Mon 2012;12:361–368.
[19] Fattovich G, Giustina G, Degos F, et al. Effectiveness of interferon alfa on incidence of hepatocellular carcinoma and decompensation in cirrhosis type C. European Concerted Action on Viral Hepatitis (EUROHEP). J Hepatol 1997;27:201–205.
[20] Morgan RL, Baack B, Smith BD, et al. Eradication of hepatitis C virus infection and the development of hepatocellular carcinoma: a meta-analysis of observational studies. Ann Intern Med 2013;158:329–337.
[21] Saab S, Hunt DR, Stone MA, et al. Timing of hepatitis C antiviral therapy in patients with advanced liver disease: a decision analysis model. Liver Transpl 2010;16:748–759.
[22] Le AK, Zhao C, Hoang JK, et al. Ethnic disparities in progression to advanced liver disease and overall survival in patients with chronic hepatitis C: impact of a sustained virological response. Aliment Pharmacol Ther 2017;46:605–616.
[23] Xie J, Xu B, Wei L, et al. Effectiveness and safety of sofosbuvir/velpatasvir/voxilaprevir as a hepatitis C virus infection salvage therapy in the real world: a systematic review and meta-analysis. Infect Dis Ther 2022;11:1661–1682.
[24] Tada T, Kurosaki M, Nakamura S, et al. Real-world clinical outcomes of sofosbuvir and velpatasvir treatment in HCV genotype 1- and 2-infected patients with decompensated cirrhosis: a nationwide multicenter study by the Japanese Red Cross Liver Study Group. J Med Virol 2021;93:6247–6256.
[25] Li M, Zhuang H, Wei L. How would China achieve WHO's target of eliminating HCV by 2030? Expert Rev Anti Infect Ther 2019;17:763–773.

[26] Rein DB, Smith BD, Wittenborn JS, et al. The cost-effectiveness of birth-cohort screening for hepatitis C antibody in U.S. primary care settings. Ann Intern Med 2012;156:263–270.
[27] Heffernan A, Ma Y, Nayagam S, et al. Economic and epidemiological evaluation of interventions to reduce the burden of hepatitis C in Yunnan province, China. PLoS One 2021;16:e0245288.
[28] Saeed YA, Phoon A, Bielecki JM, et al. A systematic review and meta-analysis of health utilities in patients with chronic hepatitis C. Value Health 2020;23:127–137.
[29] Zhao Z, Chu M, Guo Y, et al. Feasibility of hepatitis C elimination in China: from epidemiology, natural history, and intervention perspectives. Front Microbiol 2022;13:884598.
[30] Mei X, Lu H. Prevalence, diagnosis, and treatment of hepatitis C in Mainland China. Glob Health Med 2021;3:270–275.
[31] Al MJ. Cost-effectiveness acceptability curves revisited. Pharmacoeconomics 2013;31:93–100.
[32] Bonneux L, Birnie E. The discount rate in the economic evaluation of prevention: a thought experiment. J Epidemiol Community Health 2001;55:123–125.
[33] Tang W, Tao Y, Fajardo E, et al. Diagnostic accuracy of point-of-care HCV viral load assays for HCV diagnosis: a systematic review and meta-analysis. Diagnostics (Basel) 2022;12:1255.
[34] Simmons B, Saleem J, Hill A, et al. Risk of late relapse or reinfection with hepatitis C virus after achieving a sustained virological response: a systematic review and meta-analysis. Clin Infect Dis 2016;62:683–694.
[35] Modin L, Arshad A, Wilkes B, et al. Epidemiology and natural history of hepatitis C virus infection among children and young people. J Hepatol 2019;70:371–378.

Journal of Hepatology, Volume *6*

Supplemental information

Universal screening for HCV infection in China: An effectiveness and cost-effectiveness analysis

Hua Zhou, Mengxia Yan, Datian Che, and Bin Wu

Universal screening for HCV infection in China: An effectiveness and cost-effectiveness analysis

Hua Zhou, Mengxia Yan, Datian Che, Bin Wu

Table of contents

Table S1 2

Table S2. 2

Table S3. 3

Table S4. 4

Supplementary references 5

Table S1: chronic hepatitis C prevalence and population distribution according to age[1–3].

	Estimated Prevalence	Population distribution
<5 years	0.006136158	0.059738982
5-9 years	0.011715877	0.060795336
10-14 years	0.013303903	0.058791425
15-19 years	0.013311699	0.058179866
20-24 years	0.013320962	0.062222495
25-29 years	0.013606482	0.073278142
30-34 years	0.01394847	0.089365186
35-39 years	0.014203694	0.06764186
40-44 years	0.014442049	0.070192224
45-49 years	0.014593455	0.086600454
50-54 years	0.014924339	0.084757947
55-59 years	0.015225713	0.066009365
60-64 years	0.01541528	0.054072897
65-69 years	0.015538108	0.050188477
70-74 years	0.015575276	0.029066774
75-79 years	0.015777422	0.017924958
80+ years	0.016365674	0.018319479
All age	0.0138	1

Table S2: Disease stage distribution according to age.

	<18 years	18-39 years	40-59 years	>=60 years
Fibrosis F0	0.662	0.58391	0.23032	0.076995
Fibrosis F1	0.246	0.27113	0.58391	0.166105
Fibrosis F2	0.071	0.10115	0.27113	0.211645
Fibrosis F3	0.021	0.03055	0.10115	0.22273
Fibrosis F4	0.00001	0.01191	0.03055	0.31904

Table S3: Averted clinical complications in different strategies per 1000 population*.

Strategy	CC	DC	HCC	Excess mortality
Status quo (Reference strategy)	Not applicable	Not applicable	Not applicable	Not applicable
Strategy 1: Screening 18-49 years	273.9(95% UI:186.6 to 354.3)	148(95% UI:98.1 to 198.2)	123.6(95% UI:80.6 to 175.4)	1.76(95% UI:1.2 to 2.27)
Strategy 2: Screening 18-59 years	349.6(95% UI:238.3 to 452.3)	181(95% UI:120.1 to 242.7)	150(95% UI:97.7 to 211.9)	2.3(95% UI:1.59 to 2.96)
Strategy 3: Screening 18-69 years	384.5(95% UI:262.3 to 496.8)	204.8(95% UI:136.1 to 274.1)	167.2(95% UI:109.2 to 235.9)	2.71(95% UI:1.86 to 3.48)
Strategy 4: Screening 18-80 years	398(95% UI:271.3 to 515)	213(95% UI:141.5 to 285.1)	172.8(95% UI:112.9 to 243.4)	2.93(95% UI:2 to 3.78)
Strategy 5: Screening 12-80 years	442.4(95% UI:302.3 to 570.4)	240.1(95% UI:159.6 to 320.5)	196.3(95% UI:128.1 to 276.6)	3.21(95% UI:2.19 to 4.12)
Strategy 6: Screening 3-80 years	495(95% UI:339 to 636)	273.5(95% UI:182.9 to 363.3)	225.5(95% UI:147.4 to 317.7)	3.53(95% UI:2.42 to 4.52)

* Comparing with reference strategy (Status quo).

Table S4: Cost-effectiveness of seven screening strategies in China.

Strategy	cost($)	QALY	LY	ICER($/QALY)*
Status quo (Reference strategy)	19.05 (95% UI: 11.4 to 31.06)	16.506 (95% UI: 12.28 to 1.922)	40.041 (95% UI: 40.019 to 40.062)	NA
Strategy 1: Screening 18-49 years	72.06 (95% UI: 53.92 to 89.42)	16.514 (95% UI: 12.285 to 21.938)	40.072 (95% UI: 40.053 to 40.08)	7868 (95% UI: 3282 to 21655)
Strategy 2: Screening 18-59 years	90.76 (95% UI: 67.59 to 112.22)	16.516 (95% UI: 12.286 to 21.94)	40.075 (95% UI: 40.055 to 40.091)	9687 (95% UI: 3690 to 24288)
Strategy 3: Screening 18-69 years	104.06 (95% UI: 77.11 to 128.7)	16.517 (95% UI: 12.287 to 21.942)	40.077 (95% UI: 40.057 to 40.093)	8191 (95% UI: 3979 to 23056)
Strategy 4: Screening 18-80 years	111.19 (95% UI: 82.29 to 137.69)	16.518 (95% UI: 12.288 to 21.943)	40.077 (95% UI: 40 to 40.094)	7724 (95% UI: 4196 to 23644)
Strategy 5: Screening 12-80 years	118.85 (95% UI: 87.76 to 147.17)	16.519 (95% UI: 12.288 to 21.946)	40.086 (95% UI: 40.066 to 40.103)	9434 (95% UI: 3979 to 23111)
Strategy 6: Screening 3-80 years	128.18 (95% UI: 94.45 to 159.1)	16.521 (95% UI: 12.289 to 21.951)	40.101 (95% UI: 40.078 to 40.11)	9503 (95% UI: 3738 to 22566)

* Comparing with reference strategy (Status quo).

Supplementary references

[1] Assessing the cost-effectiveness of hepatitis C screening strategies in France. Journal of Hepatology 2018;69:785–92. https://doi.org/10.1016/j.jhep.2018.05.027.

[2] Kim H-L, Kim K-A, Choi GH, Jang ES, Ki M, Choi HY, et al. A cost-effectiveness study of universal screening for hepatitis C virus infection in South Korea: A societal perspective. Clin Mol Hepatol 2021;28:91–104. https://doi.org/10.3350/cmh.2021.0236.

[3] Zhou H, Lu Y, Wu B, Che D. Cost-effectiveness of Oral Regimens for Adolescents With Chronic Hepatitis C Virus Infection. Pediatr Infect Dis J 2020;39:e59–65. https://doi.org/10.1097/INF.0000000000002717.
